# Men of Color Health Awareness intervention: changes in adrenocortical activity assessed using fingernail cortisol

**DOI:** 10.3389/fpubh.2025.1526636

**Published:** 2025-07-11

**Authors:** Jerrold S. Meyer, Jeffery Markham, Lamont Scott, Luis A. Valdez, Dean E. Robinson, David R. Buchanan

**Affiliations:** ^1^Department of Psychological and Brain Sciences, University of Massachusetts, Amherst, MA, United States; ^2^Department of Health Promotion and Policy, University of Massachusetts, Amherst, MA, United States; ^3^Men of Color Health Awareness (MOCHA) Project, Springfield, MA, United States; ^4^Community Health and Prevention, Dornsife School of Public Health, Drexel University, Philadelphia, PA, United States; ^5^Department of Political Science, University of Massachusetts, Amherst, MA, United States

**Keywords:** stress, cortisol, fingernails, clinical trial, intervention, African-American, men

## Abstract

**Introduction:**

A randomized controlled trial (#NCT03745703) assessed the efficacy of a tailored stress reduction intervention (Men of Color Health Awareness; MOCHA) aimed at improving the health of middle-aged African-American men. The present paper presents an exploratory study of whether the intervention affected chronic output of the stress hormone cortisol using the novel approach of measuring cortisol accumulation in fingernail samples. Each fingernail sample was hypothesized to contain cortisol deposited over approximately a 3-month period before collection.

**Methods:**

Samples were obtained at three time points: prior to beginning of the intervention (T1; which provided an index of cortisol section during a 3-month wait-list control period); at the end of the 10-week intervention (T2); and at 12-week follow-up (T3). Self-reports of perceived stress, depression, and anxiety were collected at the same three time points plus one more: T0, which occurred 12 weeks before T1 and provided baseline psychosocial data.

**Results:**

Nail cortisol concentrations were unexpectedly higher at T2 and T3 than at T1, although only the difference at T2 reached statistical significance. Nail cortisol was not associated with any self-reported psychosocial measure at any time point.

**Discussion:**

The nail cortisol data suggest that long-term life stressors experienced by the participants may have led to a suppression of adrenal cortisol release, which was at least transiently ameliorated by the MOCHA intervention. The lack of any apparent relationships between cortisol levels and measures of perceived stress, depression, or anxiety is consistent with prior findings that endocrine responses to stress often fail to covary with subjective responses to the same stress.

## 1 Introduction

African-Americans suffer poorer overall health compared to Caucasian Americans, a difference that has been attributed, in part, to experiencing racial discrimination, economic hardships, and other major life stressors (1, 2). Such inequities highlight the need for stress-reduction policies and programs aimed at improving the health of this population group. One such program is Men of Color Health Awareness (MOCHA), a community health education program launched in 2010 in Springfield, Massachusetts. Since its inception, MOCHA has helped many African-American and Latino men improve their physical and psychological well-being as demonstrated by a number of different metrics (accessed 6/1/23).^1^

After 4 years of operation, the MOCHA steering committee invited researchers from the University of Massachusetts to assist them in validating the MOCHA program using rigorous scientific methods. This objective led to the development of MOCHA Moving Forward, a randomized controlled trial (RCT; trial registration # NCT03745703)^2^ to assess the MOCHA program’s efficacy in reducing stress as well as chronic disease risk factors such as obesity and high blood pressure in low/no-income, middle-aged African-American men (3). RCT endpoints consisted of a set of psychological, physiological, and neuroendocrine measures. For the neuroendocrine component of the trial, we chose to assess chronic output of the stress-sensitive hormone cortisol (CORT), the end-product of the hypothalamic–pituitary-adrenocortical (HPA) axis (4). Assessing chronic rather than short-term HPA activity, which is typically measured using salivary CORT levels (5), was deemed advantageous for the present study because of the susceptibility of salivary CORT to factors such as diurnal rhythmicity and day-to-day changes in participant mood and perceived stress.

A large body of research has demonstrated that circulating CORT enters and accumulates over time in growing, keratin-containing tissues such as scalp hair and nails. As a result, hair or nail CORT content provide a biomarker of long-term changes in CORT output that may result from stressful life experiences (6–9). For example, Mlili and coworkers (10) recently reviewed evidence for hair CORT as a biomarker of sleep quality and sleep-associated disorders. Hair CORT may also be used to demonstrate interactions with other steroid hormones, as shown by a significant hair CORT by hair testosterone interaction (i.e., low CORT and high testosterone) predicting increased aggressive behavior in adolescents (11). Several studies have examined the relationship between hair and salivary CORT levels. This research has helped validate the hair CORT approach by demonstrating a significant positive correlation between hair CORT and the area-under-the curve of diurnal salivary CORT (12, 13). However, a recent longitudinal study of undergraduate dental students showed that salivary and hair CORT levels do not always change in parallel. While the male students showed a moderate, but still significant, reduction in salivary CORT from the first to the second sample collected 3 months later, hair CORT showed a much greater relative reduction over the same interval (14). Another potential limitation of the hair CORT methodology is that it seems to be most appropriate for assessing HPA axis changes when the stress is still ongoing at the time of sample collection (15).

Recent studies have applied the hair CORT approach to evaluating the effects of various interventions on HPA activity. Some of these studies have reported significant intervention-related reductions in hair CORT, whereas others have failed to find such reductions despite positive effects of the intervention on perceived stress or other psychological variables. For example, decreases in hair CORT were found following a stress-reduction intervention in war-affected adolescents (16), mindfulness-based interventions in university workers (17) and in 7- to 8-year-old children (18), a stress management program in health professionals and students (19), a cognitive-behavioral intervention in prenatally depressed women (20), and a trauma-informed care program for youth welfare employees (21). In contrast, hair CORT was either unaffected or increased in studies of stress mindfulness training in police officers (22), a mindfulness- and compassion-based intervention targeted against parental burnout (23), an acceptance and commitment therapy intervention in patients with inflammatory bowel diseases (24), a holistic stress management program in patients with major depressive disorder (25), a cognitive-behavioral stress management intervention in Latina breast cancer survivors (26), or a Pythagorean self-awareness intervention applied to primary school children (27) or civil servants (28) in Crete.

A significant limitation of using hair to determine long-term CORT accumulation is lack of sample availability under certain conditions. These conditions include individuals who are bald, shave their head, or have closely cropped hair, groups that may have other hairstyles that make hair collection difficult, and populations that are resistant to providing hair samples for ethnic, cultural, or religious reasons. Of particular relevance here is that hair sampling from African-American men and women has sometimes been challenging because of either hair styling issues or various ethnic/cultural concerns (29). When hair sampling is a limiting factor, collection of fingernails can potentially serve as an alternative approach (30, 31). However, the literature on stress-related changes in nail CORT is still quite limited compared to the literature on hair CORT and stress. Notably, we are not aware of any published studies using nail CORT levels to assess the hormonal effects of a stress-reduction intervention.

The present manuscript describes an exploratory analysis of changes in fingernail CORT concentrations associated with the MOCHA Moving Forward RCT. Nail sampling was chosen instead of hair sampling because a substantial proportion of the study population had little or no hair available for collection. Nail samples for CORT measurement were obtained at three time points, each separated by a period of 12 weeks. T1 was prior to beginning of the intervention, which provided an estimate of average CORT output during the 3-month wait-list control sample; T2 was 1 week after the end of the intervention; and T3 was at a 12-week follow-up time point. Self-reports of perceived stress, depression, and anxiety were collected at the same three time points plus one more: T0, which occurred 12 weeks before T1 and provided baseline psychosocial data. We tested three primary hypotheses. The first hypothesis was that nail CORT levels would be significantly reduced at T2 and/or T3 compared to T1 as a result of the MOCHA intervention. The predicted direction of change was based on (a) the knowledge that chronic stress such as that experienced by the study population (see Discussion) most commonly leads to elevated circulating CORT (4), and (b) the results of a recent review and meta-analysis of 58 previous studies showing reduced CORT levels overall following stress-reduction interventions (32). CORT levels were most likely to be reduced by interventions involving relaxation, mindfulness, or meditation. The second hypothesis was that nail CORT would be significantly correlated with subjective stress, depression, and/or anxiety at one or more time points. Although large-scale studies have generally not found a significant relationship between psychological stress indicators and long-term HPA activity measured using hair CORT [for example (33)], most of those studies examined White populations, allowing for the possibility that a highly-stressed minority population might show a positive relationship between nail CORT and subjective stress markers. The third hypothesis was that measures of subjective stress and/or psychological variables would predict subsequent nail CORT levels. We reasoned that because the CORT content of a fingernail sample reflects adrenocortical activity over a period of several months prior to the time of sampling, psychological measures obtained before that time might show a stronger relationship to CORT levels than the same measures obtained concurrently with sample collection.

## 2 Materials and methods

### 2.1 Research objectives and research design

The MOCHA Moving Forward research project was designed to determine the effects of a health promotion intervention aimed at reducing chronic disease risk among low-income, middle-aged (35–65 years) African-American men. One aim of the project was to compare two different versions of the intervention: MOCHA Original and MOCHA Stories Matter. The original MOCHA program (i.e., MOCHA Original) was an outgrowth of community-driven concerns about health disparities plaguing the community. This grassroots effort involved a series of community focus groups and collaboration among a number of local health and human service agencies that eventually resulted in the development of a 10-week program curriculum. Participants met once a week for a 2-h guided discussion session, and they additionally attended two 60-min aerobic exercise sessions each week. Four of the discussion sessions focused on social issues facing African-American men (e.g., gender stereotypes, coping with anger, etc.) and six sessions focused on general health promotion topics such as diet and exercise. MOCHA Original had been running for 4 years before the university researchers were invited to conduct a formal scientific evaluation of the program outcomes.

The university researchers and the 10-member MOCHA Steering Committee, all long-term African-American male residents, collaborated for 2 years on two small pilot projects to build trust and reach consensus on the research objectives and methods described herein. The primary outcome of interest was stress reduction, based on increasing interest in Minority Stress Models and the role of chronic stress in fostering a range of unhealthy behaviors such as smoking, drinking, illicit drug consumption, and overeating (34). Because hypertension and obesity are also linked to increased mortality (35), hypothesized secondary outcomes of the intervention were reduced blood pressure and body mass index (BMI).

One outcome of these pilot projects was a decision to strengthen the quality and potentially the effectiveness of the MOCHA program by adding narrative strategies for communicating key health concepts and information. There is strong evidence that culture-centered narrative strategies are more effective in producing positive health effects than didactic presentations (36). Specifically, the revised program (called MOCHA Stories Matter) covered the same content as MOCHA Original but added the strategies and formats used in digital story-telling. Digital stories are videos that synthesize digital image, voiceover recording of a self-scripted story, music, and text to document personal experiences (37). This approach has previously been used in many different settings to address a variety of health issues (38–40). Table 1 compares the two versions of the weekly MOCHA curriculum [see also Valdez et al. (3) for more details].

**Table 1:** Comparison of the weekly MOCHA original and MOCHA stories matter curricula.

MOCHA Original		MOCHA Stories Matter	
WK	Title	WK	Title
1	Introduction and Overview	1	Introduction and Overview
2	Healthy Manhood: Unpacking the Man Box	2	Man(hood) Enough: Black Masculinity Across the Life Course
3	Let us Get Physical: Food, Nutrition, and Fitness – A Lifestyle	3	Grub but Hold the Fixin’s: Purchasing and Preparing Nutritious Foods
4	Stop the Pain: Breaking the Cycle of Violence	4	Be Easy: Peace Not Violence
5	The Fight Within: Stress, Mental, and Emotional Health	5	Hakuna Matata: Coping with Trauma and Loss
6	Reproductive Health, Parenting, and Relationships	6	Better Halves and Better Dads: Healthy Relationships and Fatherhood
7	Settings Goals: Chronic Disease and Sexual Health Topics	7	Too Blessed to be Stressed: Faith and Spirituality as Resilience
8	Substance Abuse	8	Substance Use, Addiction, and Recovery
9	Social Determinants: Health Disparities, Causes, and Cures	9	Family Finances: Household Budgeting, and Post-life Planning
10	Becoming Role Models and Agents of Change	10	Resistance is Not Futile: Movement Building for Social Change

The study was conducted as a wait-list control, cross-over RCT (registration #NCT03745703). Potential participants were recruited through community outreach, primarily through tabling at large community events serving the African-American community (e.g., Stone Soul picnic, Jazz Festival, etc.) in Springfield Massachusetts (3, 41). Contact information was collected and interested parties were invited to an open enrollment meeting at a pre-scheduled date, time, and location. At the open enrollment meeting, the research team presented an overview of the purpose and procedures of the research, including the eight elements of the informed consent process. A written Letter of Consent was reviewed, questions asked and answered, and potential enrollees invited to enlist. Those who volunteered to participate were then asked to complete a baseline battery of tests. In the cross-over research design, after completing the baseline questionnaire, the participants entered the wait-list control condition at a time designated as T0. Twelve weeks later (T1), having completed the 3-month wait-list control period, participants were invited back to complete the pre-test, be randomly assigned to one of two intervention groups, and begin the intervention. Participants were eligible to receive a stipend of up to $300 for their involvement in the study. Funds were distributed in five increments: $25 for completing the baseline questionnaire, $40 for completing the pre-test, $100 for attending all 10 training sessions, $60 for completing the post-test, and $75 for completing the 3-month follow-up data collection. The full project period ran from June 2016 to July 2021.

### 2.2 Psychological, physiological, and neuroendocrine measures

Overall, the project had three specific aims: (1) to determine if MOCHA was effective in reducing stress, (2) to determine if Stories Matter (the revised MOCHA intervention that added digital storytelling to document the participants’ personal experiences) was more effective than MOCHA Original, and (3) if found to have an effect, to determine which independent variables were modified by the intervention most significantly in explaining any observed reductions in stress. Data collection consisted of three types: a self-report survey questionnaire; physiological measures of blood pressure and BMI (collected by an independent third-party public health nurse); and a neuroendocrine measure, specifically fingernail CORT levels which provide an index of chronic HPA activity. Participants were excluded from the trial if (a) they had a systolic blood pressure reading of 180 mm Hg or higher at initial measurement, and/or (b) they checked any items on a pre-screening Physical Activity Readiness Questionnaire that would have precluded them from engaging in MOCHA-related physical activities. The battery of variables addressed in the survey questionnaire included: standard demographic information, the short-form Depression, Anxiety and Stress Scale [DASS-21; (42)], six individual psychological scales, and six social, institutional and structural variables. The DASS-21 has been widely used for clinical and nonclinical populations and has been validated across multiple cultures and ethnicities (43–45). Demographic variables included age, race/ethnicity, education, and source of income (which was used to determine employment status). The psychological variables included scales to assess social support, social isolation, community connectedness, diet and exercise; the six social measures examined financial troubles, food insecurity, encounters with the criminal justice system, gender stereotypes, ageism, and racism as potential sources of stress. Only the DASS and nail CORT data were used for the purposes of this paper. Other findings are reported elsewhere (3).

### 2.3 Nail collection, processing, and analysis

Participants were instructed to let their fingernails grow for a period of 2–3 weeks prior to sample collection. Additional instructions were to remove any nail polish at least 1 day before nail sampling and to wash and dry one’s hands just before collection. Finally, participants were asked to clip approximately 2 mm of nail from all 10 fingers and to place the clippings in a clean plastic storage bag. Fingernail samples were obtained at three time points: T1 (just before the beginning of the intervention), T2 (1 week after the end of the 10-week intervention, which corresponded to 12 weeks after T1), and T3 (at 12-week follow-up). However, because of interference from the COVID pandemic along with other factors beyond our control, there was substantial attrition leading to reduced numbers of nail samples across the three time points. Thus, we obtained 98 samples at T1, 57 samples at T2, and 34 samples at T3. Although there is not yet a consensus regarding the time course of CORT deposition in fingernails (5), the results of several pharmacological studies along with the known rate of nail growth suggest that fingernail samples accumulate circulating CORT over approximately 3 to 3.5 months (i.e., 12–14 weeks) prior to sample collection [see discussion in (7)]. Based on a 3-month (12-week) incorporation period, the T1 sample assessed the period when participants were in the wait-list control condition, T2 assessed a time period corresponding to the 10-week stress intervention along with 1 week pre-intervention and 1 week post-intervention, and T3 assessed the 12-week period after the end of the intervention.

Nails were stored at room temperature in their collection bags for up to several weeks before being transferred to the processing laboratory where they were stored at −20°C until later processing and analysis. Nails were processed according to the methods described in Gettler et al. (46, 47). Briefly, each sample was weighed, washed twice with isopropanol to remove external contaminants, air-dried, ground to a fine powder using a bead mill, and extracted with methanol. Methanol extracts were dried using a vacuum evaporator, reconstituted in assay buffer, spin-filtered to remove any residual particulate matter, and then assayed in duplicate along with standards and quality controls using the Arbor Assays DetectX ELISA kit (Arbor Assays, Ann Arbor, MI). This kit has been used in previous studies from our lab (46, 47) and has additionally been validated for fingernail use by Bendinskas et al. (30). Assay readouts were converted to pg CORT per mg sample weight. Intra- and inter-assay coefficients of variation (CVs) for this assay are both <10%.

### 2.4 Statistical analyses

Nail CORT values were first examined for outliers to be eliminated from subsequent statistical analysis. Using one of the standard approaches applied in many CORT stress studies, we designated outliers to be values that exceeded 3 times the standard deviation (SD) from the sample mean. We next used the Shapiro–Wilk test to determine whether the remaining data were normally distributed. This was done because hair CORT datasets are commonly right-skewed, and we reasoned that the same might be true for nail samples. Because the test showed a non-normal distribution (W = 0.342, *p* < 0.001), we performed a log10 transformation on the data and used the log values in all subsequent statistical analyses involving nail CORT. Note, however, that non-transformed data were used for presentation purposes.

Initial hypothesis testing was performed using a 1-way repeated measures ANOVA to determine whether there was an overall change in nail CORT concentrations over time. A significant main effect of time was followed by paired t-tests to determine whether nail CORT concentrations either at T2 or at T3 differed significantly from the concentrations at T1. Additionally, effect sizes for significant t-test results were determined by calculating Hedge’s g values along with 95% confidence intervals (CIs). Next, Pearson product–moment correlations tested whether there were any relationships between nail CORT at a given time point (T1, T2, or T3) and total DASS score, DASS depression score, DASS anxiety score, or DASS stress score obtained at the same time. Lastly, because of the retrospective nature of nail CORT levels due to accumulation of circulating CORT over time (see section 2.3.), we were interested in whether any of the psychological metrics obtained at a particular time point were predictive of subsequent nail CORT concentrations. To answer this question, we used linear regression analyses with total DASS score, DASS depression, DASS anxiety, or DASS stress at T0, T1, or T2 as the independent variables, with the dependent variable being nail CORT at T1, T2, and T3, respectively, (e.g., does total DASS score at T0 predict nail CORT at T1). All statistical analyses were performed using GraphPad Prism (GraphPad Software, San Diego, CA) except for Hedge’s g effect sizes, which were determined using the on-line calculator at https://effect-size-calculator.herokuapp.com/#paired-samples-t-test.

## 3 Results

Demographic information on the participants who provided usable nail CORT values at each time point (including participants from both versions of the MOCHA intervention) is provided in Table 2. This information shows that the study population largely self-identified as African-American, was generally unemployed, and overall was of relatively low educational attainment. No attempt was made to separate individuals who were unemployed because of retirement from a previous job from those who were unemployed despite being of typical working age. Approximately 20% of the initial participant pool self-identified as Latinx; however, given the study’s focus on African-American men, the data from those individuals were excluded from the present analysis. Because of the substantial participant attrition over time, we tested whether any of the demographic characteristics of the participants who provided nail samples changed from T1 to T3. The results indicated no statistically significant changes between any of the time points for any of the demographic variables.

**Table 2:** Demographic characteristics of the participants at each sampling time^a^.

Characteristic^b^	% (N) at T1 Total *N* = 96	%(N) at T2 Total *N* = 55	%(N) at T3 Total *N* = 33
Mean age (range, SD)	53.4 (28–75, 11.2)	56.6 (29–78, 11.2)	54.5 (29–75, 11.3)
Gender (% male)	100 (96)	100 (55)	100 (33)
Race/Ethnicity^c^			
African-American	86.8 (66)	93.3 (42)	82.1 (23)
Other	13.2 (10)	6.7 (3)	17.9 (5)
Education^d^			
Less than high school graduate	14.3 (11)	11.4 (5)	14.8 (4)
High school graduate	32.5 (25)	25 (11)	14.8 (4)
Certificate/training completion	9.1 (7)	20.45 (9)	3.7 (1)
Some college	23.4 (8)	29.55 (13)	29.6 (8)
Trade school or associates degree	9.1 (7)	6.8 (3)	22.2 (6)
Undergraduate degree	7.8 (6)	4.55 (2)	7.4 (2)
Graduate degree	3.9 (3)	2.3 (1)	7.4 (2)
Employment			
Full-time	10 (8)	8.9 (4)	10.7 (3)
Part-time	7.5 (6)	8.9 (4)	10.7 (3)
Unemployed	82.5 (66)	82.2 (37)	78.6 (22)

a Statistical analysis revealed no significant changes within any of the demographic variables across the three time points.

b 16/96 participants at T1, 10/55 at T2, and 5/33 at T3 declined to provide demographic information. Note that percentages shown in the table are based on the number of participants who provided the specified demographic information at each time point rather than the total number of participants sampled.

c For the Race/Ethnicity category, 4 participants did not respond to this question at T1. Participants listed as Other mainly self-identified as biracial or mixed race.

d For the Education category, 3 participants did not respond to this question at T1, 1 participant at T2, and 1 participant at T3.

There were no statistically significant differences in nail CORT between the two versions of the MOCHA intervention; therefore, the data were combined for all subsequent analyses. The initial 189 nail CORT values yielded an overall mean of 22.6 pg./mg with a standard deviation (SD) of 74.4 pg./mg. In accordance with common practice, we determined that any participant’s nail CORT that exceeded 3 times the SD from the sample mean (i.e., 22.6 + 223.2 = 246.8 pg./mg) would be considered an outlier and eliminated from further statistical analysis. This led to the elimination of two values from T1, two values from T2, and one value from T3, leaving a new total of 184 nail CORT values (96 at T1, 55 at T2, and 33 at T3). Descriptive statistics of the nail CORT concentrations at each time point are shown in Table 3.

**Table 3:** Descriptive statistics for nail cortisol concentrations at each time point.

Nail Cortisol (pg/mg)	T1	T2	T3
Mean ± SEM (N)	10.9 ± 2.4 (96)	13.3 ± 4.5 (55)	11.2 ± 4.5 (33)
Median (range)	3.4 (0.2–170.6)	5.1 (1.3–237.6)	4.5 (0.7–146.4)

**Figure 1:**
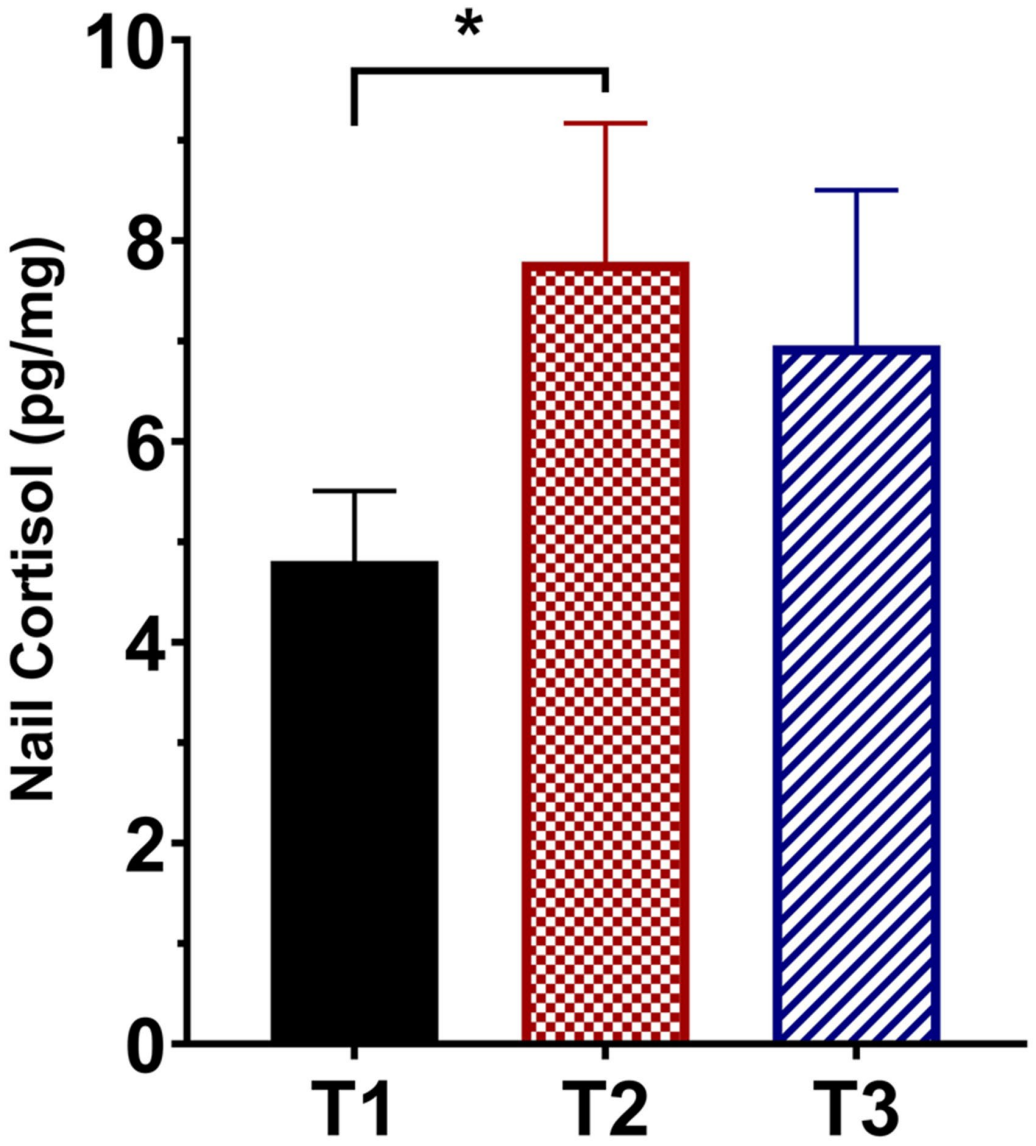
Mean (± SEM) fingernail cortisol concentrations from participants who provided usable samples for all three sampling times (T1, pre-intervention; T2, at the end of the 10-week intervention; T3, at 12-week follow-up; *N* = 31). *denotes *p* = 0.014 compared to T1.

Figure 1 presents the mean ± SEM of the untransformed nail CORT levels from the 31 participants who had usable samples at all three time points (two participants had usable samples at T3 but not at T1 and/or T2 either because the sample was not provided, or it was determined to be an outlier). We performed a repeated measures ANOVA on the log-transformed data to assess possible changes in nail CORT levels over time. The analysis revealed a significant effect of time on log nail CORT levels (*F* = 4.150, df = 2, 60, *p* = 0.025). This was followed by paired *t*-tests using the data from the same 31 participants to determine whether log nail CORT at either T2 or T3 differed significantly from the T1 CORT levels. The t-test comparing T2 with T1 was highly significant (*t* = 2.591, df = 30, *p* = 0.014; see Figure 1), with a Hedge’s g effect size of 0.625 (medium effect size) and a 95% CI of 0.124–1.149. In contrast, the comparison of T3 vs. T1, while in the same direction as T2 vs. T1, failed to reach statistical significance (*t* = 1.430, df = 30, *p* = 0.163). Similar significant results for T2 vs. T1 were obtained using the data from 54 participants who provided samples at both time points (one participant provided a nail sample at T2 but not T1; *t* = 2.567, df = 53, *p* = 0.013). For that comparison, the Hedge’s g effect size was 0.320 (small effect size) with a 95% CI of −0.069–0.717.

Two different statistical approaches were used to determine whether there were any relationships between log nail CORT values and psychological DASS scores. First, Pearson product–moment correlation analyses found no relationships between log nail cortisol values at T1, T2, and T3 to DASS scores obtained at the same time point (Table 4). Second, linear regression analyses found that DASS scores at T0, T1, and T2 failed to predict log nail CORT values measured 3 months later at T1, T2, and T3, respectively. Table 5 contains the results of the simplest multiple regression model testing main effects only. Similar non-significant results were obtained when interaction terms were included in the model (not shown).

**Table 4:** Pearson product–moment correlations of log nail cortisol with DASS scores at each time point.

Time of sampling	Log cortisol vs. DASS Dep	Log cortisol vs. DASS Anx	Log cortisol vs. DASS Str	Log cortisol vs. DASS Sum
T1	0.071	−0.019	0.084	0.050
T2	0.070	0.064	0.067	0.070
T3	0.143	0.042	−0.001	0.063

Dep, Depression subscale score; Anx, Anxiety subscale score; Str, Stress subscale score; Sum, Sum of all subscale scores.

**Table 5:** Linear regression analyses of DASS subscale values at Times 0, 1, and 2 predicting subsequent log nail cortisol levels at Times 1, 2, and 3.

Variable (Times)	*β*	SE	*p*
DASS Dep (T0-T1)	0.0295	0.0194	0.132
DASS Dep (T1-T2)	0.0031	0.0254	0.903
DASS Dep (T2-T3)	0.0323	0.0452	0.480
DASS Anx (T0-T1)	−0.0011	0.0184	0.950
DASS Anx (T1-T2)	−0.0026	0.0228	0.906
DASS Anx (T2-T3)	0.0282	0.0428	0.514
DASS Str (T0-T1)	−0.0194	0.0191	0.313
DASS Str (T1-T2)	0.0148	0.0245	0.550
DASS Str (T2-T3)	−0.0067	0.0430	0.876

Dep, Depression subscale score; Anx, Anxiety subscale score; Str, Stress subscale score.

## 4 Discussion

Using repeated fingernail CORT measures over time, the present study sought to determine (1) whether a stress-reduction intervention in low/no-income, middle-aged, African-American men significantly affected chronic activity of the HPA axis, and (2) whether changes in such activity were related to subjective measures of depression, anxiety, and stress. In contrast to our predictions, nail CORT levels were elevated when comparing post- to pre-intervention samples. Moreover, nail CORT was unrelated to any of the subjective measures obtained at any of the experimental time points.

A possible explanation for the present results relates to the dynamics of adrenocortical responsiveness under chronic versus acute stress conditions. Acute stressors typically cause transient elevations in adrenal CORT release (4). In contrast, prolonged stress exposure yields a more complicated picture. The most common result again is elevated CORT, as mentioned earlier; however, some studies of chronic stress have reported either unchanged or even decreased CORT output (48). Most relevant for the present results are those studies that reported hypocortisolism (i.e., chronically reduced CORT) following prolonged stress. Although hypocortisolism has most often been linked to the stress-related disorders fibromyalgia, chronic fatigue syndrome, and post-traumatic stress disorder, it can also occur under other conditions involving ongoing stress (49). For example, a recent Australian study of Indigenous young adults found a negative association between fingernail CORT concentrations and the number of stressful life events that had occurred over the previous 6 months [i.e., lower fingernail CORT as a function of more stressful events; (50)]. Lam and coworkers (51) additionally reported a relationship between greater lifetime stress exposure and a blunted salivary CORT response to an acute psychosocial stressor. A review and meta-analysis by Miller and colleagues (48) suggested that long-lasting stress may cause an initial hyperactivity of the HPA axis that, over time, can transition to a state of hypoactivity. From surveying the participants in the present study, it is clear they were living for many years under highly stressful conditions resulting from poverty, poor access to health care, racism, and other factors (3, 52). Other researchers have documented that African-American men experience more discrimination, racism-related stress, poverty, and crime than other studied populations (53). We hypothesize that over time, the conditions experienced by the current group of participants led to a hypoactive HPA axis that manifested in relatively low nail CORT levels. Reduced stress and enhanced health brought about by the MOCHA intervention, therefore, reversed this chronic hypoactive state as shown by the significant increase in nail CORT at T2 compared to T1. On the other hand, this effect was sufficiently diminished by T3 that it no longer reached statistical significance. It is possible that the lack of a significant CORT difference at T3 resulted from attrition of participants who were among the positive responders at T2. Perhaps some of the individuals who were positively impacted by the intervention did not feel the need to continue in the clinical trial since the intervention had ended and the T3 time point was a follow-up assessment. Alternatively, the lack of a significant CORT difference between T1 and T3 might indicate that the neuroendocrine response to the MOCHA intervention was not long-lasting. Further research is needed to determine whether the 10-week MOCHA program by itself is sufficient to produce long-term benefits, or if periodic “refresher” sessions might be required to sustain the benefits. On the other hand, it is possible that lasting stress reduction and health improvement in African-American men will not be achieved until society implements a systemic reduction in these individuals’ stressful living and working conditions.

Several previous studies of stress-reduction interventions have demonstrated that ameliorating dysregulated HPA axis function can lead to elevated CORT levels measured in various ways. Examples include an 8-week yoga intervention in women with fibromyalgia that led to an increase in diurnal salivary CORT area-under-the-curve (54), a 10-week attachment-based intervention with toddlers in foster care showing atypically low morning salivary CORT was associated with an increased CORT response to a caregiver-separation challenge (55), and an 8-week stress coping intervention in adolescent Syrian refugees that led to increased hair CORT levels in a subset of individuals with pre-existing CORT hyposecretion (16). Taken together, these findings support our hypothesis that the observed pattern of fingernail CORT changes may be related to a pre-existing adrenocortical hypoactivity that was normalized by the MOCHA intervention.

The second major finding of the present study, that nail CORT concentrations were unrelated to either subjective stress or symptoms of anxiety or depression, was not totally unexpected despite being contrary to our initial hypotheses. This is because the relatively small number of prior studies on this issue have yielded inconsistent results. For example, Wu and colleagues (56) found a significant positive correlation between perceived stress and fingernail CORT in samples obtained 15 days later from Chinese medical students. In contrast, no significant relationship between perceived stress and fingernail CORT was reported for middle-aged Japanese workers (57), adolescent and young adult males (including cisgender, genderqueer, and nonbinary individuals) (58), low-income African-American high school students (59), or Australian young adults [both Indigenous and non-Indigenous; (50)]. Furthermore, a recent detailed analysis relating subjective stress to hair CORT in young German adults found that most of the variance in hair CORT levels can be attributed to factors other than the amount of stress perceived by the participants (60). Importantly, the present study attempted to relate nail CORT to a measure of subjective stress both concurrently (nail CORT values measured at the same time as the subjective stress indicator) and retrospectively (nail CORT values measured at 3 months after the subjective stress indicator). The overall negative findings, therefore, support the idea that the mechanisms underlying HPA axis responses to stress are not strongly coordinated with the various subjective responses manifested as perceived stress or changes in mood such as depression or anxiety (61, 62).

A distributed neural network composed mainly of the prefrontal cortex, hippocampus, amygdala, and hypothalamus is responsible for coordinating both the subjective and physiological (including hormonal) responses to stress (63). Nevertheless, it appears that within a given individual, the output of the subjective component of this network may differ in magnitude from the output of the physiological component under stressful conditions. In other words, a particular stressor could elicit a large increase in perceived stress but a smaller increase in CORT secretion in one individual, whereas a different individual might show a small increase in perceived stress but a large increase in CORT secretion in response to the same stressor. The existence of this disparity supports the value of measuring both subjective and physiological outcome measures in stress intervention studies.

The present study is unique in at least two respects. It is, to our knowledge, the first study to report fingernail CORT levels in an adult African-American population. The mean T1 (pre-intervention) CORT concentration of 10.9 pg./mg is notably lower than the mean CORT concentration of 21.1 pg./mg obtained in adolescent African-American students using similar processing and analytical methods (59). This difference could be related to several factors, including the age of the participants, the mixture of both males and females in the earlier study versus males only in the present study, and other variables with unknown contributions to nail CORT accumulation. Also worth noting is that least some of the previously published studies on non-African-American populations found lower mean nail CORT levels than the mean levels reported in this study (8). Just as African-Americans generally have higher levels of hair CORT than other racial/ethnic groups [for example, see (64)], the same situation may pertain to nail CORT.

The second novel feature of the present study involves the use of nail instead of hair CORT measurements to assess changes in chronic CORT output following a stress reduction intervention. This is significant because, as mentioned in the Introduction, participants in stress studies sometimes either do not have sufficient scalp hair for sampling or are unwilling for personal or ethnic/cultural reasons to provide a hair sample. Fingernail CORT measurement is an emerging tool for stress researchers, as shown by a gradually increasing number of studies using this approach to assess long-term HPA activity. Recent work has validated this methodology by demonstrating significant correlations between fingernail CORT levels and either salivary or hair CORT (30, 65, 66). Furthermore, fingernail CORT studies have yielded results similar to earlier findings with salivary or hair CORT with respect to the time course of CORT changes during pregnancy and the postpartum period (67) as well as elevated CORT in individuals with major depressive disorder compared to healthy controls (68, 69). Considering the unanticipated increase in nail CORT following the present intervention, more research is needed to determine the conditions under which nail CORT levels are increased, decreased, or unaffected under various conditions, including stress-reduction interventions.

Several limitations of the present study should be noted. First, the generalizability of the results is limited by the fact that the study participants were all low/no-income, middle-aged, African-American men. Participants were selected according to those criteria by design because the intervention used in the study was intended specifically for that target population. Nevertheless, other populations may respond differently to a stress-reduction intervention targeted to those groups. Second, we relied solely on nail CORT levels to assess intervention-related changes in adrenal CORT output. Although accumulation of CORT in scalp hair or nails is believed to provide a reliable index of integrated circulating CORT over long time periods (8, 9), it sheds no light on potential changes in HPA axis dynamics including the CORT awakening response, CORT diurnal rhythm, and CORT responses to acute stressors that can be studied by means of multiple saliva sampling. However, attempting to investigate any of these system parameters using salivary CORT would have been extremely challenging given the difficulties encountered with respect to both recruiting African-American men into scientific investigations and maintaining communication with the study population. Third, we cannot be certain that the CORT measured in the fingernail samples was deposited over the hypothesized 3-month time period. However, we need to emphasize that this is currently a limitation of all studies measuring CORT or any other substance in nail samples. The fourth limitation is the substantial attrition in participants over the course of the study. We surmise that a number of different factors may have contributed to this attrition. First, it appears that continued participation in the study over a 9-month time period required significant internal motivation to devote the time and effort to improving the individual’s health beyond the monetary compensation provided and the opportunity to contribute to a scientific investigation. It appears that this motivation was not sufficiently strong for many of the participants who began the study, especially since the later time points for nail collection occurred after the intervention had concluded. It may be that participant “readiness to change” is needed to obtain high retention rates in a stress-reduction intervention, similar to the situation with substance misuse interventions (70). If sufficient internal motivation is not present in some of the participants, greater long-term retention might still be obtained by structuring the participant stipends so that sufficiently large stipends are paid at the later phases of the clinical trial. A second factor concerns the difficulty encountered in maintaining contact with each person over the course of their participation in the study. Because the intervention targeted a highly marginalized population, many participants lacked permanent housing, regular access to a telephone, and/or available transportation to the study site. All of these factors led to difficulties either reaching participants for follow-up or enabling participants to keep a follow-up appointment. We recommend that future studies of this kind of population obtain more information about each participant’s social network (e.g., family, friends, and colleagues) and their contact information. Such information could potentially facilitate the ability of researchers to keep track of study participants over time. Lastly, the project was hugely impacted by the COVID-19 pandemic, which began at a time of ongoing participant recruitment and data collection. The ensuing social restrictions further disrupted our ability to communicate and meet with participants. In addition, it adversely affected the participants’ social support network that had previously facilitated their commitment to the project.

In conclusion, the MOCHA Moving Forward RCT found a significant increase in fingernail CORT concentrations measured shortly following the intervention (T2) compared to the pre-intervention time point (T1). This increase persisted until the next sampling time, 3 months later (T3), although the difference compared to T1 was no longer statistically significant. Based on the dynamics of adrenocortical responsiveness to stress, these findings suggest that on-going life stress experienced by African-American men may have caused a suppression of chronic CORT output that was at least temporarily alleviated by the MOCHA intervention. However, because of the acknowledged limitations of this exploratory study, additional research is needed to confirm the present finding. Lastly, the lack of any significant relationship between nail CORT and psychological indicators of stress and mood is consistent with prior research showing that neuroendocrine responses to stress can be disconnected from the subjective responses to the same stress.

## Data Availability

The raw data supporting the conclusions of this article will be made available by the authors, without undue reservation.

## Footnotes

^1^ https://mochaspringfield.org/about

^2^ https://clinicaltrials.gov/study/NCT03745703
